# Cucurbit[6]uril *p*-xylylenediammonium diiodide deca­hydrate inclusion complex

**DOI:** 10.1107/S1600536808018412

**Published:** 2008-06-21

**Authors:** Wei-Hao Huang, Peter Y. Zavalij, Lyle Isaacs

**Affiliations:** a Department of Chemistry and Biochemistry, University of Maryland, College Park, MD 20742, USA

## Abstract

The title inclusion complex, C_36_H_36_N_24_O_12_·C_8_H_14_N_2_^2+^·2I^−^·10H_2_O, displays a large ellipsoidal deformation of the cucurbit[6]uril (CB[6]) skeleton upon complex formation. The benzene ring of the cation is rotationally disordered between two orientations in a ratio of 3:1. The solvent H_2_O mol­ecules form a hydrogen-bonded network by inter­action with the carbonyl groups of CB[6] and the I^−^ counterions. The crystal studied exhibited non-merohedral twinning. Both CB[6] and the cation are centrosymmetric.

## Related literature

For related literature, see: Bush *et al.* (2005 ▶); Freeman *et al.* (1981 ▶); Freeman (1984 ▶); Henning *et al.* (2007 ▶); Huang *et al.* (2007 ▶); Ko *et al.* (2007 ▶); Lagona *et al.* (2005 ▶); Liu *et al.* (2005 ▶); Marquez *et al.* (2004 ▶); Moon & Kaifer (2004 ▶); Rauwald & Scherman (2008 ▶); Rekharsky *et al.* (2008 ▶); Samsonenko *et al.* (2002 ▶); Wheate *et al.* (2006 ▶).
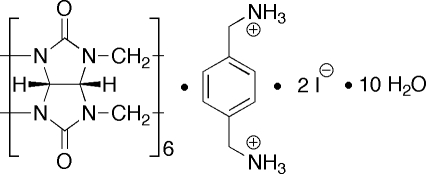


## Experimental

### 

#### Crystal data

C_36_H_36_N_24_O_12_·C_8_H_14_N_2_^2+^·2I^−^·10H_2_O
*M* *_r_* = 1569.06
Monoclinic, 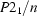

*a* = 11.9987 (9) Å
*b* = 15.9520 (12) Å
*c* = 15.0517 (11) Å
β = 92.8520 (10)°
*V* = 2877.4 (4) Å^3^
*Z* = 2
Mo *K*α radiation
μ = 1.20 mm^−1^
*T* = 220 (2) K
0.20 × 0.10 × 0.07 mm

#### Data collection

Bruker SMART 1000 three-circle diffractometer
Absorption correction: multi-scan (*SADABS*; Sheldrick, 1996 ▶) *T*_min_ = 0.798, *T*_max_ = 0.914
19804 measured reflections
6606 independent reflections
5363 reflections with *I* > 2σ(*I*)
*R*_int_ = 0.027

#### Refinement

*R*[*F* ^2^ > 2σ(*F* ^2^)] = 0.043
*wR*(*F* ^2^) = 0.083
*S* = 1.00
6606 reflections
475 parameters
33 restraints
H atoms treated by a mixture of independent and constrained refinement
Δρ_max_ = 1.68 e Å^−3^
Δρ_min_ = −0.83 e Å^−3^

### 

Data collection: *SMART* (Bruker, 1999 ▶); cell refinement: *SAINT* (Bruker, 1999 ▶); data reduction: *SAINT*; program(s) used to solve structure: *SHELXS97* (Sheldrick, 2008 ▶); program(s) used to refine structure: *SHELXL97* (Sheldrick, 2008 ▶); molecular graphics: *SHELXTL* (Sheldrick, 2008 ▶); software used to prepare material for publication: *SHELXL97*.

## Supplementary Material

Crystal structure: contains datablocks I, global. DOI: 10.1107/S1600536808018412/cv2420sup1.cif

Structure factors: contains datablocks I. DOI: 10.1107/S1600536808018412/cv2420Isup2.hkl

Additional supplementary materials:  crystallographic information; 3D view; checkCIF report

## Figures and Tables

**Table 1:** Hydrogen-bond geometry (Å, °)

*D*—H⋯*A*	*D*—H	H⋯*A*	*D*⋯*A*	*D*—H⋯*A*
N1—H1*A*⋯O23^i^	0.90	2.25	2.861 (5)	124
N1—H1*A*⋯O33^i^	0.90	2.27	3.040 (5)	143
N1—H1*B*⋯O1*W*	0.90	1.99	2.833 (5)	155
N1—H1*B*⋯O3*W*^ii^	0.90	2.39	2.922 (6)	118
N1—H1*C*⋯O13^i^	0.90	2.01	2.910 (5)	175
O1*W*—H11*W*⋯O34^ii^	0.843 (19)	2.19 (3)	2.837 (4)	133 (4)
O1*W*—H12*W*⋯O2*W*	0.852 (19)	1.80 (2)	2.655 (6)	175 (6)
O2*W*—H21*W*⋯O5*W*	0.870 (18)	2.21 (3)	2.973 (6)	146 (5)
O2*W*—H22*W*⋯O24	0.85 (2)	1.91 (3)	2.712 (5)	157 (7)
O3*W*—H31*W*⋯O5*W*	0.870 (18)	1.97 (2)	2.820 (7)	167 (4)
O3*W*—H32*W*⋯O34	0.892 (19)	2.23 (4)	2.847 (5)	126 (4)
O4*W*—H41*W*⋯O14	0.831 (19)	2.51 (5)	3.198 (5)	141 (6)
O4*W*—H42*W*⋯O1*W*	0.835 (19)	2.11 (5)	2.823 (6)	143 (7)
O5*W*—H51*W*⋯I1^iii^	0.82 (2)	2.80 (3)	3.601 (4)	167 (6)
O5*W*—H52*W*⋯I1	0.824 (19)	2.80 (4)	3.573 (4)	157 (7)

Symmetry codes: (i) 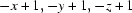; (ii) 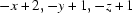; (iii) 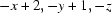.

**Fig. 1.**
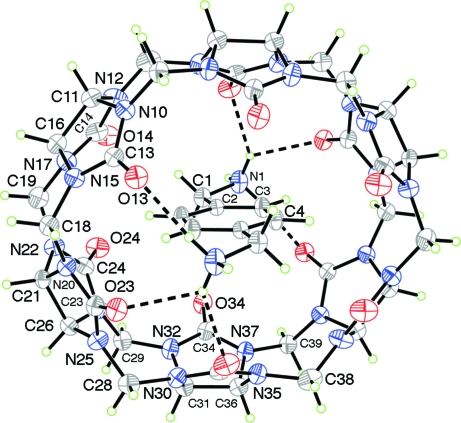
Molecular structure of complex (I) showing atom-labeling scheme and displacement ellipsoids at the 30% probability level. The unlabelled atoms are related with the labelled ones by symmetry operation (1-x, 1-y, 1-z). The iodide counterions and solvating water molecules are omitted for clarity.

**Fig. 2.**
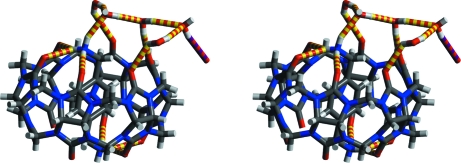
Cross-eyed stereoview of the structure of complex (I) in the crystal showing the chain of H2O molecules H-bonded to the ureidyl C═O portal of CB[6] and terminated by H-bonding to iodide.

**Fig. 3.**
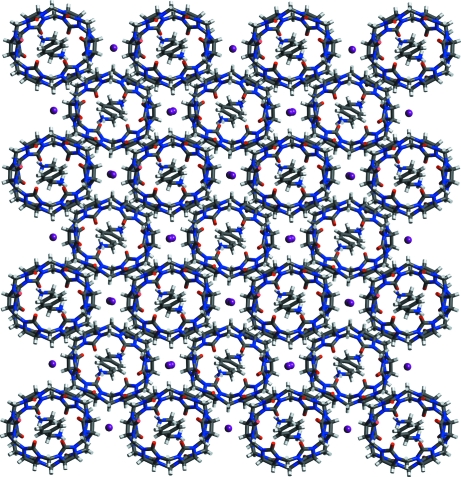
Illustration of the packing of complex (I) in the bc-plane of the crystal. Color coding: C, gray; H, white; N, blue; O, red; I, purple; H-bonds, red-yellow striped.

## supplementary crystallographic information

### Comment

In supramolecular chemistry of the cucurbit[*n*]uril family of molecular containers has expanded dramatically in recent years with the preparation of a homologous series of hosts (*e.g.* CB[*n*], n = 5, 6, 7, 8, 10), inverted CB[*n*], CB[*n*] derivatives and analogues, and most recently nor-seco-CB[*n*] (Lagona *et al.*, 2005). The high affinity and high selectivity of CB[*n*]-type receptors toward their guests (Liu *et al.*, 2005), most notably organic ammonium ions, has lead to their use in a variety of applications including molecular machines (Ko *et al.* 2007), drug delivery (Wheate *et al.* 2006), chemical sensors (Henning *et al.* 2007), self-assembled macromolecules (Moon & Kaifer, 2004; Rauwald & Scherman 2008), and recognition of biomolecules (Bush *et al.*, 2005; Rekharsky *et al.* 2008).

The polycyclic nature of CB[*n*] molecular containers suggests that this class of molecules might be relatively rigid and have difficulty responding to the size and shape of its guests. There is evidence, however, that CB[*n*] molecular containers may undergo substantial deformations both in transition states for ingression and egression of guests and in the ground state of the corresponding host–guest complexes (Huang *et al.*, 2007; Marquez *et al.*, 2004; Samsonenko *et al.*, 2002). This paper reports the X-ray crystal structure of complex (I) which exhibits such a deformation.

The asymmetric unit of complex (I) comprises one half of a CB[6] molecule and one half of a *p*-xylylenediammonium cation both disposed about a center of inversion, an iodide counterion and five water molecules. The majority of the structural features of complex (I) are as expected based on its molecular structure, but several deserve some comment. For example, the *p*-xylylenediammonium ions are held in the cavity of CB[6] by H-bonds to its ureidyl C═O portals (Fig. 1 and Table 1). The solvating H_2_O molecules form a cap on the complex by H-bonding to CB[6], the diammonium ion, themselves, and finally terminated by H-bonding to the I^-^ counterion (Fig. 2). Complex (I) packs in the crystal by formation of a square array in the *bc* plane (Fig. 3).

Most interesting is the substantial ellipsoidal deformation observed in complex (I). We quantify this distortion for complex (I) as 0.88 Å (non-bonded C—C range 9.852–10.730 Å) by determining the distances between opposing C-atoms along the equator of the molecule as suggested previously by Samsonenko (Samsonenko *et al.*, 2002). Although this ellipsoidal deformation is modest relative to those previously reported for complexes of CB[6] and other CB[*n*]-type receptors, taken together the results highlight the ability of CB[*n*]-type receptors to respond to the size and shape of their guests.

### Experimental

Complex (I) was prepared by mixing cucurbit[6]uril with 1,4-xylylenediamine dihydrochloride in water according to the literature procedure (Freeman *et al.*, 1981; Freeman, 1984; Liu *et al.*, 2005) followed by the addition of KI. Single crystals suitable for structure determination were obtained by allowing the aqueous solution of complex (I) to stand at room temperature for several days.

### Refinement

The N- and C-bound H atoms were included in the riding-model approximation with N—H = 0.90 Å and C—H = 0.98 to 0.99 Å, and with *U*_iso_(H) = 1.5*U*_eq_(N) and *U*_iso_(H) = 1.2*U*_eq_(C). The water H atoms were refined with soft restraints [O—H = 0.84 (3) Å, H—O—H = 105 (2)° in a riding model approximation, with *U*_iso_(H) = 1.5*U*_eq_(O). The benzene ring of the cation is rotationally disordered between two orientations in a ratio of 3:1. The highest residual peak (1.68 e Å^-3^) is located 0.95 Å from I1 atom and is due to either partial disorder of I1 atom or truncation effect. The crystal studied is non-merohedral twin consisting of two components (domains). The twinning law is 180° rotation around 100 reciprocal direction with approximate 7:1 domain ratio.

### Crystal data

**Table:**

C_36_H_36_N_24_O_12_·C_8_H_14_N_2_^2+^·2I^–^·10H_2_O	*F*_000_ = 1596
*M**_r_* = 1569.06	*D*_x_ = 1.811 Mg m^−^^3^
Monoclinic, *P*2_1_/*n*	Mo *K*α radiation λ = 0.71073 Å
Hall symbol: -P 2yn	Cell parameters from 6752 reflections
*a* = 11.9987 (9) Å	θ = 2.1–25.0º
*b* = 15.9520 (12) Å	µ = 1.20 mm^−^^1^
*c* = 15.0517 (11) Å	*T* = 220 (2) K
β = 92.8520 (10)º	Prism, colourless
*V* = 2877.4 (4) Å^3^	0.21 × 0.10 × 0.08 mm
*Z* = 2	

### Data collection

**Table:**

Bruker SMART1000 three-circle diffractometer	6606 independent reflections
Radiation source: fine-focus sealed tube	5363 reflections with *I* > 2σ(*I*)
Monochromator: graphite	*R*_int_ = 0.027
Detector resolution: 8.33 pixels mm^-1^	θ_max_ = 25.0º
*T* = 220(2) K	θ_min_ = 2.6º
ω scans	*h* = −14→14
Absorption correction: multi-scan(SADABS; Sheldrick, 1996)	*k* = 0→18
*T*_min_ = 0.798, *T*_max_ = 0.914	*l* = 0→17
19804 measured reflections	

### Refinement

**Table:**

Refinement on *F*^2^	Secondary atom site location: difference Fourier map
Least-squares matrix: full	Hydrogen site location: inferred from neighbouring sites
*R*[*F*^2^ > 2σ(*F*^2^)] = 0.043	H atoms treated by a mixture of independent and constrained refinement
*wR*(*F*^2^) = 0.083	*w* = 1/[σ^2^(*F*_o_^2^) + (0.01*P*)^2^ + 9.45*P*], *P* = (max(*F*_o_^2^,0) + 2*F*_c_^2^)/3
*S* = 1.00	(Δ/σ)_max_ = 0.001
6606 reflections	Δρ_max_ = 1.68 e Å^−^^3^
475 parameters	Δρ_min_ = −0.83 e Å^−^^3^
33 restraints	Extinction correction: none
Primary atom site location: structure-invariant direct methods	

### Special details

**Table:**

Geometry. All e.s.d.'s (except the e.s.d. in the dihedral angle between two l.s. planes) are estimated using the full covariance matrix. The cell e.s.d.'s are taken into account individually in the estimation of e.s.d.'s in distances, angles and torsion angles; correlations between e.s.d.'s in cell parameters are only used when they are defined by crystal symmetry. An approximate (isotropic) treatment of cell e.s.d.'s is used for estimating e.s.d.'s involving l.s. planes.
Refinement. Refinement of *F*^2^ against ALL reflections. The weighted *R*-factor *wR* and goodness of fit *S* are based on *F*^2^, conventional *R*-factors *R* are based on *F*, with *F* set to zero for negative *F*^2^. The threshold expression of *F*^2^ > σ(*F*^2^) is used only for calculating *R*-factors(gt) *etc*. and is not relevant to the choice of reflections for refinement. *R*-factors based on *F*^2^ are statistically about twice as large as those based on *F*, and *R*- factors based on ALL data will be even larger.The crystal is non-merohedral twin in about 7:1 ratio with 180° rotation around 100 reciprocal axis.

### Fractional atomic coordinates and isotropic or equivalent isotropic displacement parameters (Å^2^)

**Table:**

	*x*	*y*	*z*	*U*_iso_*/*U*_eq_	Occ. (<1)
N1	0.7930 (3)	0.5633 (2)	0.5760 (2)	0.0507 (10)	
H1A	0.7603	0.5953	0.6166	0.076*	
H1B	0.8618	0.5832	0.5673	0.076*	
H1C	0.7979	0.5101	0.5958	0.076*	
C1	0.7258 (3)	0.5656 (3)	0.4919 (3)	0.0443 (10)	
H1D	0.7641	0.5331	0.4473	0.053*	
H1E	0.7210	0.6237	0.4710	0.053*	
C2	0.6097 (3)	0.5316 (2)	0.4984 (3)	0.0338 (8)	0.75 (2)
C3	0.5731 (6)	0.4915 (8)	0.5724 (4)	0.042 (2)	0.75 (2)
H3	0.6213	0.4862	0.6232	0.050*	0.75 (2)
C4	0.4658 (7)	0.4587 (8)	0.5731 (4)	0.042 (2)	0.75 (2)
H4	0.4440	0.4293	0.6235	0.051*	0.75 (2)
C2A	0.6097 (3)	0.5316 (2)	0.4984 (3)	0.0338 (8)	0.25 (2)
C3A	0.5490 (14)	0.538 (3)	0.5730 (11)	0.048 (7)	0.25 (2)
H3A	0.5817	0.5623	0.6248	0.058*	0.25 (2)
C4A	0.4395 (15)	0.509 (3)	0.5729 (14)	0.055 (8)	0.25 (2)
H4A	0.3983	0.5175	0.6236	0.065*	0.25 (2)
N10	0.3002 (3)	0.7278 (2)	0.4134 (2)	0.0404 (8)	
C11	0.3824 (3)	0.7816 (3)	0.3748 (3)	0.0419 (10)	
H11	0.3561	0.8402	0.3694	0.050*	
N12	0.4926 (3)	0.7764 (2)	0.4189 (3)	0.0514 (10)	
C13	0.2665 (3)	0.6641 (3)	0.3575 (3)	0.0378 (9)	
O13	0.1943 (2)	0.61228 (18)	0.3720 (2)	0.0477 (7)	
C14	0.5705 (4)	0.7472 (3)	0.3620 (3)	0.0492 (11)	
O14	0.6714 (2)	0.7448 (2)	0.3790 (2)	0.0597 (9)	
N15	0.3242 (3)	0.6691 (2)	0.2818 (2)	0.0410 (8)	
C16	0.3977 (3)	0.7412 (3)	0.2827 (3)	0.0417 (10)	
H16	0.3776	0.7804	0.2335	0.050*	
N17	0.5157 (3)	0.7200 (2)	0.2855 (2)	0.0443 (9)	
C18	0.3017 (3)	0.6167 (3)	0.2047 (3)	0.0424 (10)	
H18A	0.3035	0.6515	0.1510	0.051*	
H18B	0.2264	0.5934	0.2072	0.051*	
C19	0.5726 (4)	0.6914 (3)	0.2075 (3)	0.0525 (12)	
H19A	0.6504	0.7101	0.2134	0.063*	
H19B	0.5381	0.7189	0.1549	0.063*	
N20	0.3804 (2)	0.5487 (2)	0.1978 (2)	0.0369 (8)	
C21	0.4813 (3)	0.5542 (2)	0.1478 (3)	0.0382 (9)	
H21	0.4651	0.5740	0.0862	0.046*	
N22	0.5717 (3)	0.6010 (2)	0.1919 (2)	0.0421 (8)	
C23	0.3496 (3)	0.4674 (3)	0.2112 (3)	0.0393 (10)	
O23	0.2621 (2)	0.44348 (18)	0.2413 (2)	0.0458 (7)	
C24	0.6548 (3)	0.5501 (3)	0.2251 (3)	0.0414 (10)	
O24	0.7409 (2)	0.57396 (19)	0.2638 (2)	0.0554 (8)	
N25	0.4334 (3)	0.4166 (2)	0.1863 (2)	0.0410 (8)	
C26	0.5247 (3)	0.4628 (3)	0.1501 (3)	0.0395 (10)	
H26	0.5425	0.4424	0.0903	0.047*	
N27	0.6246 (3)	0.4686 (2)	0.2104 (2)	0.0414 (8)	
C28	0.4310 (3)	0.3270 (3)	0.1997 (3)	0.0471 (11)	
H28A	0.4655	0.2999	0.1494	0.056*	
H28B	0.3530	0.3088	0.1988	0.056*	
C29	0.7032 (3)	0.3999 (3)	0.2238 (3)	0.0474 (11)	
H29A	0.7030	0.3663	0.1693	0.057*	
H29B	0.7783	0.4232	0.2341	0.057*	
N30	0.4870 (3)	0.2973 (2)	0.2816 (2)	0.0429 (9)	
C31	0.6038 (3)	0.2749 (3)	0.2874 (3)	0.0438 (10)	
H31	0.6236	0.2381	0.2376	0.053*	
N32	0.6791 (3)	0.3455 (2)	0.2977 (2)	0.0453 (9)	
C33	0.4323 (3)	0.2741 (3)	0.3553 (3)	0.0439 (10)	
O33	0.3330 (2)	0.28222 (19)	0.3662 (2)	0.0543 (8)	
C34	0.7397 (3)	0.3437 (3)	0.3768 (3)	0.0417 (10)	
O34	0.8151 (2)	0.39229 (19)	0.4000 (2)	0.0512 (8)	
N35	0.5085 (3)	0.2381 (2)	0.4152 (3)	0.0455 (9)	
C36	0.6176 (3)	0.2293 (3)	0.3780 (3)	0.0435 (10)	
H36	0.6383	0.1698	0.3708	0.052*	
N37	0.7055 (3)	0.2765 (2)	0.4248 (2)	0.0437 (9)	
C38	0.4791 (4)	0.1889 (3)	0.4935 (4)	0.0598 (13)	
H38A	0.3984	0.1791	0.4892	0.072*	
H38B	0.5154	0.1340	0.4899	0.072*	
C39	0.7588 (3)	0.2502 (3)	0.5088 (3)	0.0442 (10)	
H39A	0.7680	0.1891	0.5076	0.053*	
H39B	0.8335	0.2751	0.5142	0.053*	
I1	0.78087 (2)	0.50257 (2)	0.010322 (19)	0.05174 (9)	
O1W	0.9837 (3)	0.6263 (3)	0.4947 (3)	0.0760 (11)	
H11W	1.035 (3)	0.593 (3)	0.512 (3)	0.114*	
H12W	0.978 (5)	0.619 (4)	0.4385 (14)	0.114*	
O2W	0.9547 (3)	0.6070 (3)	0.3199 (3)	0.0937 (14)	
H21W	0.994 (4)	0.571 (3)	0.291 (4)	0.141*	
H22W	0.891 (3)	0.584 (4)	0.310 (5)	0.141*	
O3W	1.0139 (4)	0.3995 (3)	0.3046 (3)	0.0880 (12)	
H31W	1.019 (6)	0.427 (3)	0.255 (2)	0.132*	
H32W	0.969 (4)	0.433 (3)	0.334 (4)	0.132*	
O4W	0.8962 (3)	0.7906 (3)	0.4916 (3)	0.0839 (11)	
H41W	0.8269 (16)	0.790 (4)	0.486 (5)	0.126*	
H42W	0.914 (5)	0.746 (3)	0.467 (4)	0.126*	
O5W	1.0206 (3)	0.5122 (4)	0.1607 (3)	0.0947 (14)	
H51W	1.073 (3)	0.507 (5)	0.129 (4)	0.142*	
H52W	0.965 (3)	0.495 (5)	0.133 (4)	0.142*	

### Atomic displacement parameters (Å^2^)

**Table:**

	*U*^11^	*U*^22^	*U*^33^	*U*^12^	*U*^13^	*U*^23^
N1	0.0256 (18)	0.070 (3)	0.056 (2)	−0.0157 (17)	−0.0027 (16)	0.012 (2)
C1	0.031 (2)	0.054 (2)	0.048 (3)	−0.0075 (19)	0.0007 (18)	0.005 (2)
C2	0.0290 (19)	0.038 (2)	0.034 (2)	−0.0021 (15)	0.0010 (16)	0.0013 (17)
C3	0.032 (3)	0.056 (6)	0.037 (3)	−0.004 (3)	−0.008 (2)	0.006 (3)
C4	0.036 (4)	0.054 (6)	0.037 (4)	−0.007 (4)	0.005 (3)	0.010 (3)
C2A	0.0290 (19)	0.038 (2)	0.034 (2)	−0.0021 (15)	0.0010 (16)	0.0013 (17)
C3A	0.041 (9)	0.06 (2)	0.043 (11)	0.002 (11)	0.005 (7)	−0.008 (11)
C4A	0.042 (9)	0.07 (2)	0.048 (12)	0.008 (11)	0.021 (8)	−0.007 (13)
N10	0.0284 (18)	0.048 (2)	0.045 (2)	−0.0041 (15)	0.0042 (15)	−0.0011 (17)
C11	0.029 (2)	0.042 (2)	0.054 (3)	−0.0006 (18)	0.0004 (18)	0.000 (2)
N12	0.0265 (19)	0.064 (3)	0.063 (3)	−0.0022 (17)	−0.0015 (17)	−0.022 (2)
C13	0.024 (2)	0.043 (2)	0.045 (3)	0.0019 (18)	−0.0013 (17)	0.002 (2)
O13	0.0295 (15)	0.0550 (19)	0.059 (2)	−0.0099 (14)	0.0055 (13)	0.0007 (15)
C14	0.031 (2)	0.046 (3)	0.072 (3)	−0.005 (2)	0.009 (2)	−0.004 (2)
O14	0.0255 (17)	0.066 (2)	0.088 (3)	−0.0058 (14)	0.0051 (15)	−0.0152 (18)
N15	0.0326 (19)	0.046 (2)	0.045 (2)	−0.0062 (15)	0.0072 (15)	−0.0025 (17)
C16	0.029 (2)	0.043 (2)	0.052 (3)	0.0002 (18)	0.0034 (18)	0.004 (2)
N17	0.0298 (19)	0.054 (2)	0.049 (2)	−0.0009 (16)	0.0088 (16)	−0.0014 (18)
C18	0.030 (2)	0.049 (3)	0.048 (3)	0.0032 (18)	−0.0029 (18)	0.001 (2)
C19	0.037 (2)	0.059 (3)	0.062 (3)	−0.010 (2)	0.010 (2)	0.005 (2)
N20	0.0228 (17)	0.041 (2)	0.046 (2)	0.0025 (14)	−0.0010 (14)	0.0019 (16)
C21	0.033 (2)	0.045 (2)	0.037 (2)	−0.0002 (18)	−0.0022 (18)	0.0046 (19)
N22	0.0252 (18)	0.044 (2)	0.057 (2)	−0.0023 (15)	0.0012 (15)	−0.0032 (17)
C23	0.026 (2)	0.049 (2)	0.042 (2)	0.0013 (18)	−0.0089 (18)	0.0041 (19)
O23	0.0231 (15)	0.0559 (18)	0.0581 (19)	−0.0046 (13)	0.0003 (13)	0.0063 (15)
C24	0.029 (2)	0.052 (3)	0.044 (3)	−0.0023 (19)	0.0068 (19)	−0.006 (2)
O24	0.0262 (16)	0.066 (2)	0.073 (2)	−0.0050 (14)	−0.0053 (14)	−0.0117 (17)
N25	0.0227 (17)	0.040 (2)	0.059 (2)	−0.0004 (14)	−0.0019 (15)	0.0022 (17)
C26	0.030 (2)	0.049 (2)	0.040 (2)	0.0006 (18)	−0.0002 (18)	−0.0031 (19)
N27	0.0227 (17)	0.051 (2)	0.051 (2)	0.0043 (15)	−0.0018 (15)	0.0001 (17)
C28	0.029 (2)	0.054 (3)	0.057 (3)	−0.0015 (19)	−0.0060 (19)	−0.004 (2)
C29	0.033 (2)	0.058 (3)	0.050 (3)	0.002 (2)	−0.0011 (19)	0.004 (2)
N30	0.0276 (18)	0.046 (2)	0.054 (2)	0.0014 (15)	−0.0057 (16)	0.0062 (18)
C31	0.031 (2)	0.050 (3)	0.050 (3)	0.0072 (19)	−0.0032 (18)	0.002 (2)
N32	0.0306 (19)	0.054 (2)	0.050 (2)	−0.0072 (16)	−0.0067 (16)	0.0113 (18)
C33	0.028 (2)	0.037 (2)	0.066 (3)	−0.0050 (18)	−0.003 (2)	−0.007 (2)
O33	0.0269 (17)	0.0540 (19)	0.082 (2)	−0.0067 (14)	0.0009 (15)	−0.0034 (17)
C34	0.029 (2)	0.048 (3)	0.048 (3)	0.0038 (19)	0.0001 (19)	0.006 (2)
O34	0.0310 (16)	0.061 (2)	0.061 (2)	−0.0100 (14)	−0.0080 (14)	0.0086 (16)
N35	0.0333 (19)	0.051 (2)	0.053 (2)	−0.0046 (16)	0.0019 (17)	0.0059 (18)
C36	0.033 (2)	0.047 (3)	0.051 (3)	0.0008 (19)	−0.0011 (19)	0.000 (2)
N37	0.0318 (18)	0.053 (2)	0.045 (2)	−0.0039 (16)	−0.0063 (15)	0.0077 (17)
C38	0.039 (3)	0.056 (3)	0.084 (4)	−0.005 (2)	0.002 (3)	−0.003 (3)
C39	0.033 (2)	0.051 (3)	0.048 (3)	0.0045 (18)	−0.0021 (19)	0.004 (2)
I1	0.05282 (17)	0.05554 (18)	0.04705 (16)	−0.01189 (15)	0.00454 (12)	0.00013 (16)
O1W	0.0357 (19)	0.103 (3)	0.089 (3)	−0.0053 (18)	0.0004 (19)	0.021 (3)
O2W	0.041 (2)	0.119 (4)	0.119 (4)	−0.004 (2)	−0.005 (2)	−0.004 (3)
O3W	0.074 (3)	0.091 (3)	0.099 (3)	0.006 (2)	0.010 (2)	0.002 (2)
O4W	0.071 (2)	0.082 (3)	0.100 (3)	−0.002 (2)	0.005 (2)	−0.008 (2)
O5W	0.050 (2)	0.168 (4)	0.066 (2)	0.003 (3)	0.0075 (18)	0.000 (3)

### Geometric parameters (Å, °)

**Table:**

N1—C1	1.467 (5)	C23—N25	1.359 (5)
N1—H1A	0.9000	C24—O24	1.222 (5)
N1—H1B	0.9000	C24—N27	1.364 (5)
N1—H1C	0.9000	N25—C28	1.445 (5)
C1—C2	1.503 (5)	N25—C26	1.449 (5)
C1—H1D	0.9800	C26—N27	1.470 (5)
C1—H1E	0.9800	C26—H26	0.9900
C2—C3	1.375 (6)	N27—C29	1.453 (5)
C2—C4^i^	1.380 (8)	C28—N30	1.453 (5)
C3—C4	1.391 (7)	C28—H28A	0.9800
C3—H3	0.9400	C28—H28B	0.9800
C4—C2^i^	1.380 (8)	C29—N32	1.452 (5)
C4—H4	0.9400	C29—H29A	0.9800
C3A—C4A	1.393 (11)	C29—H29B	0.9800
C3A—H3A	0.9400	N30—C33	1.368 (5)
C4A—C2A^i^	1.36 (2)	N30—C31	1.444 (5)
C4A—H4A	0.9400	C31—N32	1.447 (5)
N10—C13	1.368 (5)	C31—C36	1.547 (6)
N10—C39^i^	1.441 (5)	C31—H31	0.9900
N10—C11	1.451 (5)	N32—C34	1.364 (5)
C11—N12	1.452 (5)	C33—O33	1.218 (5)
C11—C16	1.549 (6)	C33—N35	1.377 (5)
C11—H11	0.9900	C34—O34	1.228 (5)
N12—C14	1.379 (5)	C34—N37	1.367 (5)
N12—C38^i^	1.455 (6)	N35—C36	1.455 (5)
C13—O13	1.225 (4)	N35—C38	1.474 (6)
C13—N15	1.365 (5)	C36—N37	1.449 (5)
C14—O14	1.225 (5)	C36—H36	0.9900
C14—N17	1.367 (6)	N37—C39	1.450 (5)
N15—C18	1.445 (5)	C38—N12^i^	1.455 (6)
N15—C16	1.449 (5)	C38—H38A	0.9800
C16—N17	1.453 (5)	C38—H38B	0.9800
C16—H16	0.9900	C39—N10^i^	1.441 (5)
N17—C19	1.460 (5)	C39—H39A	0.9800
C18—N20	1.446 (5)	C39—H39B	0.9800
C18—H18A	0.9800	I1—H52W	2.80 (4)
C18—H18B	0.9800	O1W—H11W	0.843 (19)
C19—N22	1.461 (5)	O1W—H12W	0.852 (19)
C19—H19A	0.9800	O2W—H21W	0.870 (18)
C19—H19B	0.9800	O2W—H22W	0.85 (2)
N20—C23	1.366 (5)	O3W—H31W	0.870 (18)
N20—C21	1.460 (5)	O3W—H32W	0.892 (19)
C21—N22	1.450 (5)	O4W—H41W	0.831 (19)
C21—C26	1.547 (5)	O4W—H42W	0.835 (19)
C21—H21	0.9900	O5W—H51W	0.82 (2)
N22—C24	1.361 (5)	O5W—H52W	0.824 (19)
C23—O23	1.225 (5)		
			
C1—N1—H1A	109.5	C24—O24—H22W	164.0 (18)
C1—N1—H1B	109.5	C23—N25—C28	122.1 (3)
H1A—N1—H1B	109.5	C23—N25—C26	112.6 (3)
C1—N1—H1C	109.5	C28—N25—C26	125.2 (3)
H1A—N1—H1C	109.5	N25—C26—N27	114.1 (3)
H1B—N1—H1C	109.5	N25—C26—C21	103.2 (3)
N1—C1—C2	113.9 (3)	N27—C26—C21	102.6 (3)
N1—C1—H1D	108.8	N25—C26—H26	112.1
C2—C1—H1D	108.8	N27—C26—H26	112.1
N1—C1—H1E	108.8	C21—C26—H26	112.1
C2—C1—H1E	108.8	C24—N27—C29	122.1 (3)
H1D—C1—H1E	107.7	C24—N27—C26	111.1 (3)
C3—C2—C4^i^	117.4 (4)	C29—N27—C26	122.8 (3)
C3—C2—C1	123.7 (4)	N25—C28—N30	115.4 (3)
C4^i^—C2—C1	118.9 (4)	N25—C28—H28A	108.4
C2—C3—C4	121.0 (5)	N30—C28—H28A	108.4
C2—C3—H3	119.5	N25—C28—H28B	108.4
C4—C3—H3	119.5	N30—C28—H28B	108.4
C2^i^—C4—C3	121.6 (5)	H28A—C28—H28B	107.5
C2^i^—C4—H4	119.2	N32—C29—N27	113.8 (3)
C3—C4—H4	119.2	N32—C29—H29A	108.8
C4A—C3A—H3A	119.6	N27—C29—H29A	108.8
C2A^i^—C4A—C3A	122.1 (13)	N32—C29—H29B	108.8
C2A^i^—C4A—H4A	119.0	N27—C29—H29B	108.8
C3A—C4A—H4A	119.0	H29A—C29—H29B	107.7
C13—N10—C39^i^	122.7 (3)	C33—N30—C31	112.8 (3)
C13—N10—C11	112.2 (3)	C33—N30—C28	123.8 (3)
C39^i^—N10—C11	123.5 (3)	C31—N30—C28	122.6 (4)
N10—C11—N12	113.9 (3)	N30—C31—N32	114.3 (3)
N10—C11—C16	103.1 (3)	N30—C31—C36	103.3 (3)
N12—C11—C16	103.8 (3)	N32—C31—C36	103.4 (3)
N10—C11—H11	111.8	N30—C31—H31	111.7
N12—C11—H11	111.8	N32—C31—H31	111.7
C16—C11—H11	111.8	C36—C31—H31	111.7
C14—N12—C11	111.5 (4)	C34—N32—C31	112.4 (3)
C14—N12—C38^i^	123.8 (4)	C34—N32—C29	124.3 (4)
C11—N12—C38^i^	123.9 (3)	C31—N32—C29	122.0 (3)
O13—C13—N15	125.5 (4)	O33—C33—N30	126.4 (4)
O13—C13—N10	125.5 (4)	O33—C33—N35	125.3 (4)
N15—C13—N10	108.9 (3)	N30—C33—N35	108.3 (3)
O14—C14—N17	126.5 (4)	O34—C34—N32	126.0 (4)
O14—C14—N12	125.0 (4)	O34—C34—N37	125.5 (4)
N17—C14—N12	108.5 (4)	N32—C34—N37	108.4 (4)
C14—O14—H41W	145.2 (12)	C34—O34—H32W	132.1 (14)
C13—N15—C18	123.7 (3)	C33—N35—C36	111.6 (4)
C13—N15—C16	111.9 (3)	C33—N35—C38	124.6 (4)
C18—N15—C16	123.7 (3)	C36—N35—C38	120.8 (3)
N15—C16—N17	114.0 (3)	N37—C36—N35	114.2 (4)
N15—C16—C11	103.8 (3)	N37—C36—C31	103.2 (3)
N17—C16—C11	103.2 (3)	N35—C36—C31	103.6 (3)
N15—C16—H16	111.7	N37—C36—H36	111.8
N17—C16—H16	111.7	N35—C36—H36	111.8
C11—C16—H16	111.7	C31—C36—H36	111.8
C14—N17—C16	112.2 (3)	C34—N37—C36	112.4 (3)
C14—N17—C19	123.4 (4)	C34—N37—C39	123.8 (3)
C16—N17—C19	122.8 (4)	C36—N37—C39	123.5 (3)
N15—C18—N20	113.2 (3)	N12^i^—C38—N35	117.8 (4)
N15—C18—H18A	108.9	N12^i^—C38—H38A	107.8
N20—C18—H18A	108.9	N35—C38—H38A	107.8
N15—C18—H18B	108.9	N12^i^—C38—H38B	107.8
N20—C18—H18B	108.9	N35—C38—H38B	107.8
H18A—C18—H18B	107.7	H38A—C38—H38B	107.2
N17—C19—N22	116.0 (4)	N10^i^—C39—N37	115.1 (3)
N17—C19—H19A	108.3	N10^i^—C39—H39A	108.5
N22—C19—H19A	108.3	N37—C39—H39A	108.5
N17—C19—H19B	108.3	N10^i^—C39—H39B	108.5
N22—C19—H19B	108.3	N37—C39—H39B	108.5
H19A—C19—H19B	107.4	H39A—C39—H39B	107.5
C23—N20—C18	121.3 (3)	H1B—O1W—H42W	97.1
C23—N20—C21	111.8 (3)	H1B—O1W—H11W	99.4
C18—N20—C21	123.6 (3)	H42W—O1W—H11W	154 (4)
N22—C21—N20	114.5 (3)	H1B—O1W—H12W	118.7
N22—C21—C26	103.4 (3)	H42W—O1W—H12W	85 (4)
N20—C21—C26	102.6 (3)	H11W—O1W—H12W	104 (3)
N22—C21—H21	111.9	H12W—O2W—H21W	120 (4)
N20—C21—H21	111.9	H12W—O2W—H22W	109 (5)
C26—C21—H21	111.9	H21W—O2W—H22W	97 (3)
C24—N22—C21	112.2 (3)	H31W—O3W—H32W	101 (3)
C24—N22—C19	122.1 (4)	H41W—O4W—H42W	103 (4)
C21—N22—C19	125.5 (3)	H31W—O5W—H21W	69.1 (15)
O23—C23—N25	125.2 (4)	H31W—O5W—H51W	113 (6)
O23—C23—N20	126.4 (4)	H21W—O5W—H51W	136 (5)
N25—C23—N20	108.4 (3)	H31W—O5W—H52W	96 (6)
O24—C24—N22	125.1 (4)	H21W—O5W—H52W	116 (5)
O24—C24—N27	125.8 (4)	H51W—O5W—H52W	107 (4)
N22—C24—N27	109.0 (3)		
			
N1—C1—C2—C3	−8.4 (9)	O23—C23—N25—C26	177.8 (4)
N1—C1—C2—C4^i^	171.1 (7)	N20—C23—N25—C26	−2.8 (5)
C4^i^—C2—C3—C4	2.9 (10)	C23—N25—C26—N27	105.7 (4)
C1—C2—C3—C4	−177.6 (5)	C28—N25—C26—N27	−71.4 (5)
C2—C3—C4—C2^i^	−3.0 (10)	C23—N25—C26—C21	−4.8 (4)
C13—N10—C11—N12	−112.2 (4)	C28—N25—C26—C21	178.1 (4)
C39^i^—N10—C11—N12	81.8 (5)	N22—C21—C26—N25	129.2 (3)
C13—N10—C11—C16	−0.4 (4)	N20—C21—C26—N25	9.8 (4)
C39^i^—N10—C11—C16	−166.4 (3)	N22—C21—C26—N27	10.4 (4)
N10—C11—N12—C14	117.8 (4)	N20—C21—C26—N27	−108.9 (3)
C16—C11—N12—C14	6.4 (5)	O24—C24—N27—C29	−13.1 (7)
N10—C11—N12—C38^i^	−72.2 (5)	N22—C24—N27—C29	168.9 (3)
C16—C11—N12—C38^i^	176.4 (4)	O24—C24—N27—C26	−171.2 (4)
C39^i^—N10—C13—O13	−10.4 (6)	N22—C24—N27—C26	10.7 (5)
C11—N10—C13—O13	−176.5 (4)	N25—C26—N27—C24	−124.0 (4)
C39^i^—N10—C13—N15	168.0 (3)	C21—C26—N27—C24	−13.2 (4)
C11—N10—C13—N15	1.9 (4)	N25—C26—N27—C29	78.1 (5)
C11—N12—C14—O14	172.2 (4)	C21—C26—N27—C29	−171.1 (3)
C38^i^—N12—C14—O14	2.2 (7)	C23—N25—C28—N30	−93.7 (5)
C11—N12—C14—N17	−9.3 (5)	C26—N25—C28—N30	83.1 (5)
C38^i^—N12—C14—N17	−179.3 (4)	C24—N27—C29—N32	112.2 (4)
O13—C13—N15—C18	4.8 (6)	C26—N27—C29—N32	−92.2 (5)
N10—C13—N15—C18	−173.5 (3)	N25—C28—N30—C33	102.0 (5)
O13—C13—N15—C16	175.6 (4)	N25—C28—N30—C31	−89.8 (5)
N10—C13—N15—C16	−2.7 (5)	C33—N30—C31—N32	−109.9 (4)
C13—N15—C16—N17	114.0 (4)	C28—N30—C31—N32	80.7 (5)
C18—N15—C16—N17	−75.2 (5)	C33—N30—C31—C36	1.7 (5)
C13—N15—C16—C11	2.4 (4)	C28—N30—C31—C36	−167.6 (4)
C18—N15—C16—C11	173.2 (3)	N30—C31—N32—C34	116.4 (4)
N10—C11—C16—N15	−1.1 (4)	C36—C31—N32—C34	4.8 (5)
N12—C11—C16—N15	117.9 (3)	N30—C31—N32—C29	−76.3 (5)
N10—C11—C16—N17	−120.4 (3)	C36—C31—N32—C29	172.1 (3)
N12—C11—C16—N17	−1.3 (4)	N27—C29—N32—C34	−107.2 (5)
O14—C14—N17—C16	−173.1 (4)	N27—C29—N32—C31	87.1 (5)
N12—C14—N17—C16	8.4 (5)	C31—N30—C33—O33	−176.7 (4)
O14—C14—N17—C19	−6.7 (7)	C28—N30—C33—O33	−7.5 (7)
N12—C14—N17—C19	174.8 (4)	C31—N30—C33—N35	2.1 (5)
N15—C16—N17—C14	−116.1 (4)	C28—N30—C33—N35	171.3 (4)
C11—C16—N17—C14	−4.2 (5)	C31—N32—C34—O34	174.4 (4)
N15—C16—N17—C19	77.4 (5)	C29—N32—C34—O34	7.5 (7)
C11—C16—N17—C19	−170.7 (4)	C31—N32—C34—N37	−3.2 (5)
C13—N15—C18—N20	−101.9 (4)	C29—N32—C34—N37	−170.2 (4)
C16—N15—C18—N20	88.4 (5)	O33—C33—N35—C36	173.4 (4)
C14—N17—C19—N22	106.0 (5)	N30—C33—N35—C36	−5.4 (5)
C16—N17—C19—N22	−89.0 (5)	O33—C33—N35—C38	12.9 (7)
N15—C18—N20—C23	109.8 (4)	N30—C33—N35—C38	−166.0 (4)
N15—C18—N20—C21	−92.6 (4)	C33—N35—C36—N37	117.7 (4)
C23—N20—C21—N22	−123.7 (4)	C38—N35—C36—N37	−80.9 (5)
C18—N20—C21—N22	76.8 (5)	C33—N35—C36—C31	6.2 (4)
C23—N20—C21—C26	−12.4 (4)	C38—N35—C36—C31	167.6 (4)
C18—N20—C21—C26	−171.9 (3)	N30—C31—C36—N37	−123.9 (3)
N20—C21—N22—C24	105.8 (4)	N32—C31—C36—N37	−4.5 (4)
C26—C21—N22—C24	−5.0 (4)	N30—C31—C36—N35	−4.6 (4)
N20—C21—N22—C19	−69.0 (5)	N32—C31—C36—N35	114.9 (3)
C26—C21—N22—C19	−179.8 (4)	O34—C34—N37—C36	−177.7 (4)
N17—C19—N22—C24	−91.7 (5)	N32—C34—N37—C36	−0.1 (5)
N17—C19—N22—C21	82.6 (5)	O34—C34—N37—C39	−3.8 (7)
C18—N20—C23—O23	−10.6 (7)	N32—C34—N37—C39	173.8 (4)
C21—N20—C23—O23	−170.6 (4)	N35—C36—N37—C34	−108.8 (4)
C18—N20—C23—N25	170.0 (3)	C31—C36—N37—C34	3.0 (4)
C21—N20—C23—N25	10.0 (5)	N35—C36—N37—C39	77.3 (5)
C21—N22—C24—O24	178.8 (4)	C31—C36—N37—C39	−171.0 (4)
C19—N22—C24—O24	−6.3 (7)	C33—N35—C38—N12^i^	−113.2 (5)
C21—N22—C24—N27	−3.2 (5)	C36—N35—C38—N12^i^	87.9 (5)
C19—N22—C24—N27	171.8 (4)	C34—N37—C39—N10^i^	103.4 (4)
O23—C23—N25—C28	−5.0 (6)	C36—N37—C39—N10^i^	−83.3 (5)
N20—C23—N25—C28	174.4 (4)		

Symmetry codes: (i) −*x*+1, −*y*+1, −*z*+1.

### Hydrogen-bond geometry (Å, °)

**Table:**

*D*—H···*A*	*D*—H	H···*A*	*D*···*A*	*D*—H···*A*
N1—H1A···O23^i^	0.90	2.25	2.861 (5)	124
N1—H1A···O33^i^	0.90	2.27	3.040 (5)	143
N1—H1B···O1W	0.90	1.99	2.833 (5)	155
N1—H1B···O3W^ii^	0.90	2.39	2.922 (6)	118
N1—H1C···O13^i^	0.90	2.01	2.910 (5)	175
O1W—H11W···O34^ii^	0.843 (19)	2.19 (3)	2.837 (4)	133 (4)
O1W—H12W···O2W	0.852 (19)	1.80 (2)	2.655 (6)	175 (6)
O2W—H21W···O5W	0.870 (18)	2.21 (3)	2.973 (6)	146 (5)
O2W—H22W···O24	0.85 (2)	1.91 (3)	2.712 (5)	157 (7)
O3W—H31W···O5W	0.870 (18)	1.97 (2)	2.820 (7)	167 (4)
O3W—H32W···O34	0.892 (19)	2.23 (4)	2.847 (5)	126 (4)
O4W—H41W···O14	0.831 (19)	2.51 (5)	3.198 (5)	141 (6)
O4W—H42W···O1W	0.835 (19)	2.11 (5)	2.823 (6)	143 (7)
O5W—H51W···I1^iii^	0.82 (2)	2.80 (3)	3.601 (4)	167 (6)
O5W—H52W···I1	0.824 (19)	2.80 (4)	3.573 (4)	157 (7)

Symmetry codes: (i) −*x*+1, −*y*+1, −*z*+1; (ii) −*x*+2, −*y*+1, −*z*+1; (iii) −*x*+2, −*y*+1, −*z*.
